# The Matter in Extreme Conditions instrument at the Linac Coherent Light Source

**DOI:** 10.1107/S1600577515004865

**Published:** 2015-04-21

**Authors:** Bob Nagler, Brice Arnold, Gary Bouchard, Richard F. Boyce, Richard M. Boyce, Alice Callen, Marc Campell, Ruben Curiel, Eric Galtier, Justin Garofoli, Eduardo Granados, Jerry Hastings, Greg Hays, Philip Heimann, Richard W. Lee, Despina Milathianaki, Lori Plummer, Andreas Schropp, Alex Wallace, Marc Welch, William White, Zhou Xing, Jing Yin, James Young, Ulf Zastrau, Hae Ja Lee

**Affiliations:** a Linac Coherent Light Source, SLAC National Accelerator Laboratory, 2575 Sand Hill Road, Menlo Park, CA 94025, USA

**Keywords:** FEL, high energy density science, warm dense matter, X-ray Thomson scattering, high-pressure science

## Abstract

The LCLS beam provides revolutionary capabilities for studying the transient behavior of matter in extreme conditions. The particular strength of the Matter in Extreme Conditions instrument is that it combines the unique LCLS beam with high-power optical laser beams, and a suite of dedicated diagnostics tailored for this field of science. In this paper an overview of the beamline, the capabilities of the instrumentation, and selected highlights of experiments and commissioning results are presented.

## Abstract 2

A description of the Matter in Extreme Conditions instrument at the Linac Coherent Light Source is given. Recent scientific highlights illustrate phase-contrast imaging of shock waves, X-ray Thomson scattering and X-ray diffraction of shocked materials.

## 1. Introduction   

The study of materials under extreme conditions (*i.e.* high energy density) has, in the last decade, garnered enormous scientific interest. Three reports by the National Research Council (Davidson, 2003 ▶), the National Science and Technology Council (Davidson, 2004 ▶) and the National Academy of Sciences (Turner, 2003 ▶) identified it as an area where key scientific questions needed to be answered in the new millennium.

Despite its transient character in laboratory experiments, matter in extreme conditions (MEC) is found abundantly in nature and is of high relevance in astrophysics, planetary physics and geophysics (Guillot, 1999 ▶). The high laser intensities required to create it can generate electrons with relativistic velocity distributions, an active research field in itself. The hot electrons are at the origin of various secondary sources such as proton beams, ion beams (Belyaev *et al.*, 2008 ▶) or X-rays (Murnane *et al.*, 1991 ▶). They can be used to isochorically heat matter (Hoarty *et al.*, 2007 ▶), be the spark in the fast ignition approach (Tabak *et al.*, 1994 ▶) to inertial confinement fusion or be used to model astrophysical systems (Mondal *et al.*, 2012 ▶). High pressures at modest temperature (*i.e.* around 1 eV) can be generated with energetic lasers by creating shock waves, to study shock-induced chemical reactions, equations of state, material strength, dislocation dynamics and high-strain-rate phenomena (*cf*. Kalantar *et al.*, 2005 ▶, and references therein).

The Linac Coherent Light Source (LCLS) beam (Emma *et al.*, 2010 ▶) allows for unique investigation of matter in extreme conditions using diagnostic methods such as Thomson scattering, emission and absorption spectroscopy, diffraction and phase-contrast imaging. Augmented with optical diagnostics, such as Velocity Interferometry for Any Reflector (VISAR) and Fourier Domain Interferometry (FDI), the instrument will be at the forefront of, and have a major impact on, MEC science.

## 2. Instrument overview   

### 2.1. X-ray beamline   

The MEC experimental chamber is located in hutch 6 at roughly 460 m from the end of the LCLS undulator. The X-ray beam is steered with three silicon carbide coated mirrors with incidence angle of 1.325 mrad, which reflect photon energies up to 25 keV, with a gradual decrease for higher photon energies. The mirrors have the dual function to steer the beam to the center of the MEC chamber, and spectrally and spatially filter gamma rays, bremsstrahlung and broad spectrum undulator radiation that is present on top of the free-electron laser (FEL) beam. The finite size of the mirrors and the grazing-incidence angle determine the lower photon energy limit: the higher divergence at lower photon energy tends to overfill the mirrors, increasingly reducing the beamline transmission.

Ce:YAG scintillator screens can be placed into the beam at multiple locations, to check the spatial profile and alignment of the beam. Non-destructive intensity monitors can be placed in the beam. They consist of Si_3_N_4_ foils, which back-scatter a small fraction of the beam onto X-ray photodiodes. The resulting signal gives a relative measurement of the pulse energy. Four different foil thicknesses (50 nm, 100 nm, 500 nm and 1 µm) are available, depending on the photon energy and signal level required.

The MEC hutch uses beryllium compound refractive lenses to focus the beam to the center of the target chamber. These lenses can be placed from 3.9 m to 4.4 m from the interaction region, which allows spot sizes on target from a best focus of 2 µm to a defocused spot of 150 µm. The unfocused beam size in the MEC hutch is around 1000 µm at 8 keV, which overfills the aperture of the Be lenses. A slit system is installed to reduce the beam size to match the aperture of the Be lenses. It consists of both vertical and horizontal Si_3_N_4_ slits, which do not damage under full FEL radiaton. The LCLS beam contains a component at the third harmonic of the fundamental photon frequency, with a magnitude of around 1% of the fundamental. Since this radiation can partially pass through the Si_3_N_4_ slits, a second set of slits made of tungsten is present. In addition, the MEC instrument contains two silicon harmonic rejection mirrors, which can be placed in the beam at an angle that rejects the third harmonic while still reflecting the fundamental. These mirrors offset the beam between 3 and 5 mm in the vertical direction.

The interaction region is at the center of the MEC target chamber, a cylindrical vacuum vessel with a 2 m diameter. The target chamber operates at a vacuum pressure of 10^−5^ mbar. The chamber is separated from the rest of beamline, which operates at pressures lower than 10^−8^ mbar, with a 25 µm Be window. Due to the change in divergence and absorption in the Be lenses and window, the beamline transmission up to the target chamber is highly photon energy dependent, ranging from 60% for 8 keV to less than 1% below 2.5 keV.

**Figure 1:**
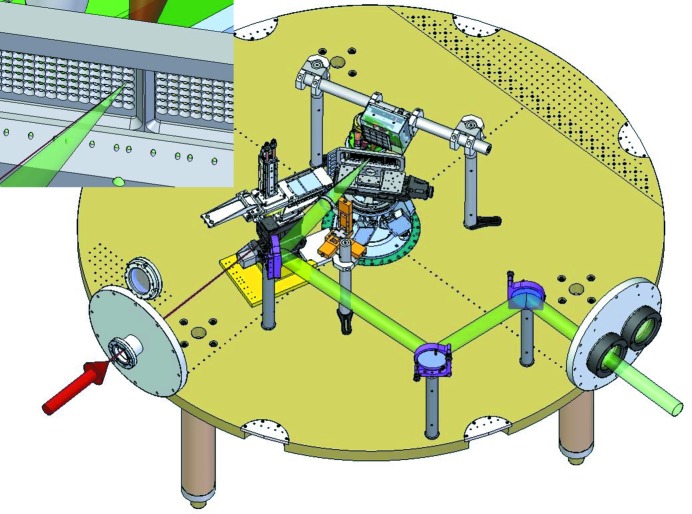
Example of the setup at MEC of a typical experiment and close-up of typical samples (inset). The target sample is in the center of the MEC vacuum chamber, on a motorized alignment stage with six degrees of freedom. The optical beam (in green) is overlapped on the sample with the LCLS X-ray beam using a motorized lens for steering. Long-distance microscopes are used to image both beams on a Ce:YAG scintillating target. An HAPG X-ray Thomson spectrometer records X-ray back-scatter. A large-area diffracton detector (CSPAD-560k) records elastic scatter for a solid angle of approximately 0.75 sr.

The large volume of the chamber provides room for multiple vacuum-compatible spectrometers and scattering diagnostics, and allows for the routing of the MEC laser beam to the interaction region in multiple configurations. It makes the MEC instrument very versatile and configurations regularly change in between experiments. Fig. 1 ▶ shows a typical setup of the MEC experimental chamber, with the beam delivery of the optical Nd:glass laser (see §2.2) and the X-ray beam, in addition to an X-ray Thomson scattering spectrometer (see also §3.2) and large-area diffraction detector (see also §3.3). In a typical experiment, the optical beam either drives a shock wave through a target or heats it to a high temperature. The shocked or heated region is then probed with the X-rays. Since the targets are typically destroyed by the optical laser beam on each shot, many targets can be mounted and positioned in the interaction region without venting the chamber. A motorized alignment stage, with six degrees of freedom, allows for a total area of 150 mm × 25 mm to mount targets.

The optical and X-ray beam are aligned and overlapped on a Ce:YAG scintillator target at the interaction point. The fluorescence signal created by the optical or X-ray laser beam can be imaged with long-distance microscopes, with a resolution of 18 µm, and overlap is achieved by steering the optical beam with a motorized focusing lens.

**Figure 2:**
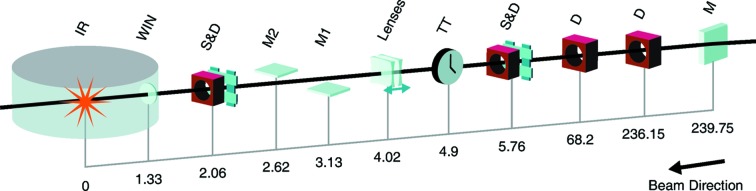
Overview of the MEC instrument layout. Distances are indicated in meters from the interaction region (IR). M is the last off-set mirror, D are non-destructive intensity and alignment diagnostics, S&D are slits and non-destructive intensity diagnostics, TT is a timetool to measure the arrival time of the Ti:S laser relative to the X-ray pulse, Lenses are beryllium compound refractive lenses, M1 and M2 are mirrors to reject the ∼1% third harmonic that is present in the LCLS beam, WIN is a 25 µm Be window, and IR is the standard interaction region (*i.e.* the center of the MEC target chamber). The interaction region is located approximately 460 m downstream of the undulator.

In Fig. 2 ▶ a schematic of the MEC beamline including the most important devices and their location is shown. Table 1 ▶ summarizes the specifications of the X-ray beamline, the optical laser and details of the hutch.

### 2.2. Optical lasers   

Compared with the other hutches at LCLS, MEC distinguishes itself by virtue of its two relatively high power and high energy optical laser systems.

#### 2.2.1. Nd:glass laser system   

The front-end of the Nd:glass laser system at MEC consists of a CW cavity operating at 1054 nm. The beam is subsequently sent through an electro-optical modulator and molded to the required pulse length and shape. The modulator has a rise time of 200 ps, and intrinsic time jitter with respect to its input trigger of 20 ps. Pulse lengths from 2 ns up to 200 ns are possible. The pulse is subsequently split into two arms, further amplified, and finally frequency-doubled to 527 nm. The maximum beam energy per arm is 25 J for pulses that are 25 ns or longer. For shorter pulses, the beams are intensity-limited to approximately 1 J ns^−1^, although this is dependent on the pulse shape. The repetition rate is determined by the cool-down time of the large-diameter glass amplifiers, which is approximately 7 min. The unfocused beam size is 40 mm. The beams are typically focused with 250 mm focal-length singlet lenses and hybrid phase plates for focal spots of 100 µm, 150 µm, 250 µm and 500 µm. Alternatively, users can bring their own focusing solution.

### 2.3. Ti:sapphire laser   

The Ti:sapphire laser at MEC has a front-end oscillator that is phase-locked to the RF of the LCLS linac to assure timing synchronization with the X-ray pulses, resulting in an arrival time jitter between the X-rays and the optical beam of approximately 150 fs RMS. The oscillator output is stretched to 150 ps and amplified in a regenerative amplifier. The output of this regenerative amplifier is compressed and sent through a cross-polarized-wave generator (XPW) to enhance the contrast and reduce the pre-pulse on the laser. The cleaned pulse is re-stretched and amplified to a final energy of 1.5 J, with a repetition rate of 5 Hz. Adaptive optics (*i.e.* a deformable mirror) are installed after the final amplifier to remove any wavefront aberrations that are present. The beam is compressed in vacuum to 50 fs and sent to the chamber with a beam diameter of 50 mm and an energy of 1 J.

Alternatively, the beam can be sent to a final amplifier that uses the arms of the MEC glass laser as a pump, increasing the energy to 7 J after compression, with a beam diameter of 85 mm. Cool-down time of the glass pump limits the repetition rate to one shot every seven minutes in this operation mode.

More detailed information about the laser, and the way it is used and synchronized with the LCLS X-ray beam, is given by Minitti *et al.* (2015 ▶).

## 3. Highlights   

### 3.1. Phase-contrast imaging and ptychography   

The ability to focus the LCLS beam down to sub-micrometer spots, and its coherent nature, allow for imaging applications with unprecedented spatial and temporal resolution. MEC has taken advantage of this capability to make phase-contrast images of shock waves going through a solid (Schropp *et al.*, 2012 ▶). For this experiment, the LCLS beam was focused with another set of Be lenses placed inside the MEC target chamber that is not part of the standard MEC beamline. The lens set has a focal length of 170 mm and focuses to a spot of nominally 85 nm (FWHM).

**Figure 3:**
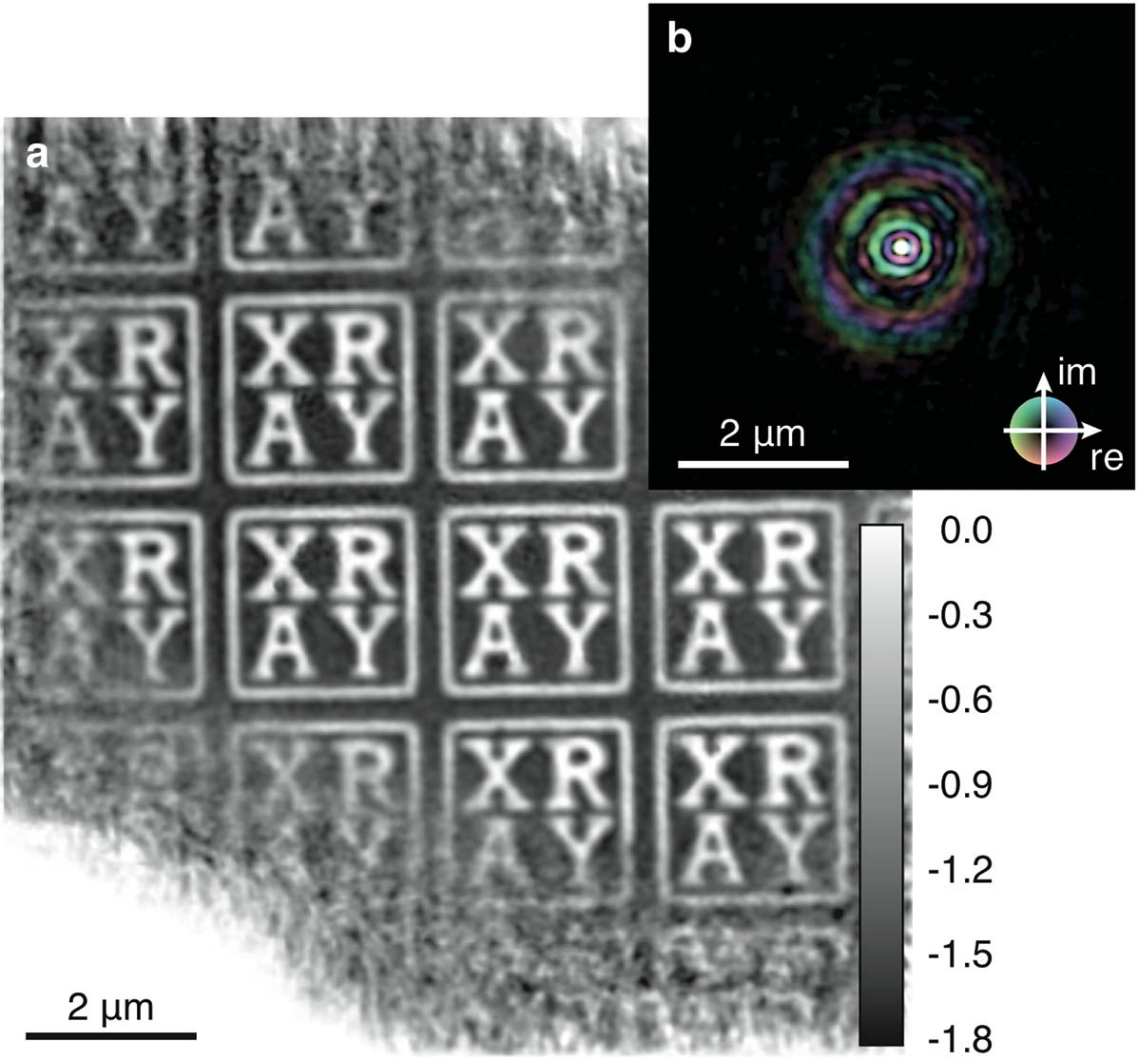
Ptychographic reconstruction of a nano-structured sample and illumination function. (*a*) Phase of the transmission function of the sample. Gray values indicate the phase shift in radians. (*b*) Complex-valued illumination function. Amplitude is encoded by brightness and phase by hue.

The characterization of this spot, which is the illumination source of the phase-contrast imaging (PCI) experiment, is highly important for a quantitative analysis of PCI data. This step was performed using scanning coherent X-ray microscopy (often also denoted by ptychography) (Rodenburg & Faulkner, 2004 ▶; Thibault *et al.*, 2009 ▶; Maiden & Rodenburg, 2009 ▶; Schropp *et al.*, 2010 ▶, 2013*a* ▶,*b* ▶). The technique is based on the scanning of a nano-structured sample through a spatially confined and coherent X-ray beam. The measurement of the far-field diffraction patterns at each scan position allows for the reconstruction of the transmission function of the sample as well as the focused LCLS beam. In Fig. 3 ▶ the results of such a ptychographic experiment are summarized. The focus size was determined to have a central spot of approximately 100 nm (FWHM).

**Figure 4:**
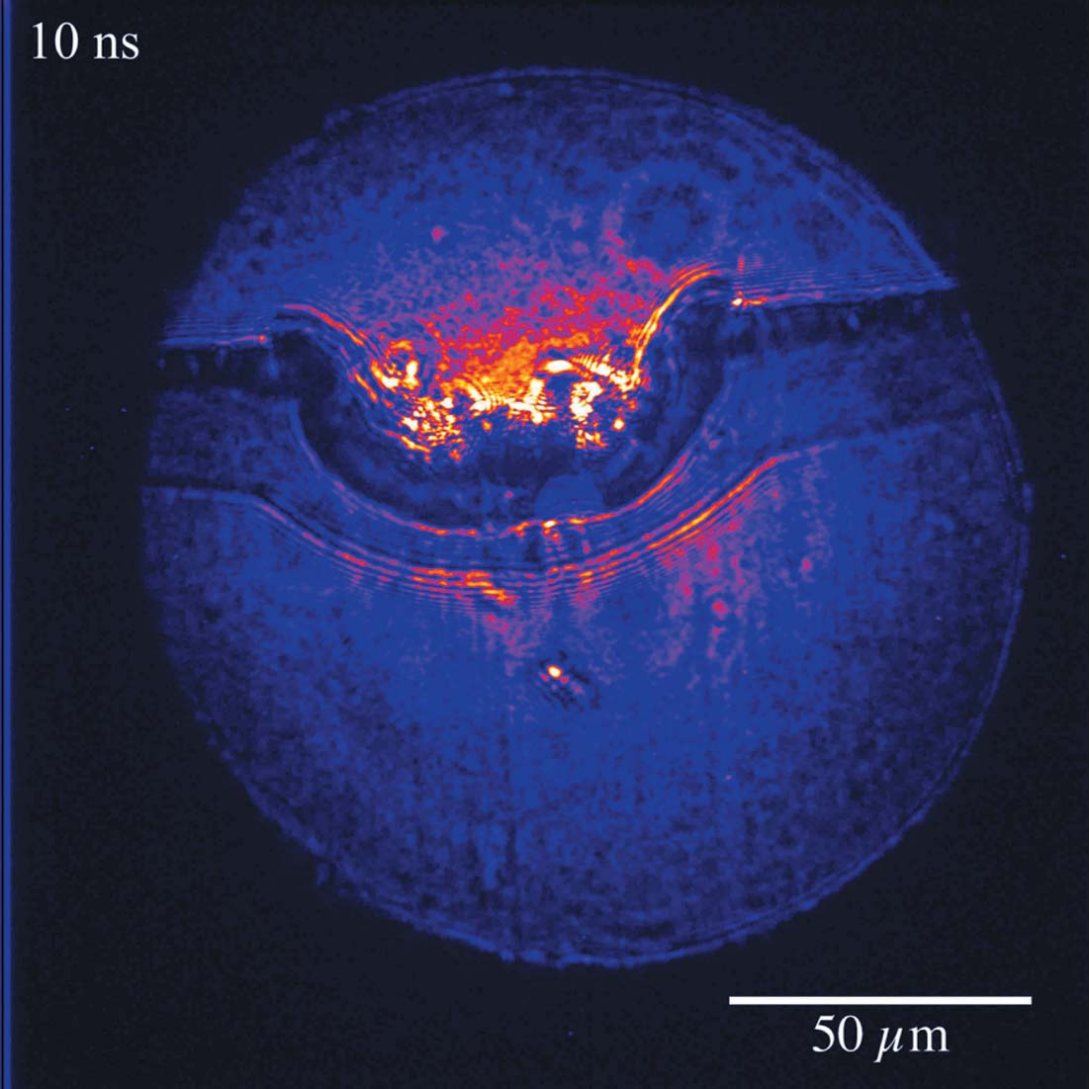
Phase-contrast image of shock propagating through an aluminium sample.

After this characterization, a sample is placed into the divergent X-ray beam at a distance of 0.2 m behind the focus. Another 3.8 m further downstream an X-ray detector is positioned in order to measure the magnified phase-contrast image of the sample. High-resolution images are obtained with sensitivity to both amplitude and phase of the X-ray transmission function of the sample. As an example, Fig. 4 ▶ shows an image of a shock wave generated by the MEC glass laser. The laser is oriented perpendicular to the X-rays and is focused on the edge of a 100 µm-thick aluminium foil. The laser is fired, and a shockwave is created when it hits the foil. After 10 ns, the X-rays arrive and capture a snap-shot of the sample. The front of the shock wave, and regions of compressed matter behind it, can clearly be distinguished with a spatial resolution better than 1 µm.

### 3.2. X-ray Thomson scattering   

In recent years, a lot of effort has gone into measuring dense matter properties at extreme conditions using X-ray Thomson scattering. The spectral analysis of the elastically and inelastically scattered X-rays has provided valuable information about electron density, temperature, ionization state and collective behavior (Glenzer *et al.*, 1999 ▶; Landen *et al.*, 2001 ▶; Gregori *et al.*, 2003 ▶; Lee *et al.*, 2009 ▶; Kritcher *et al.*, 2008 ▶; Ma *et al.*, 2014 ▶). However, experimental constraints due to the broadband uncollimated laser-produced X-ray sources (*i.e.* backlighters) have limited the experimental accuracy, which makes distinguishing among competing theoretical models difficult. MEC offers the ability to collect highly accurate data using LCLS, revolutionizing X-ray Thomson scattering as a technique to diagnose dense plasmas.

**Figure 5:**
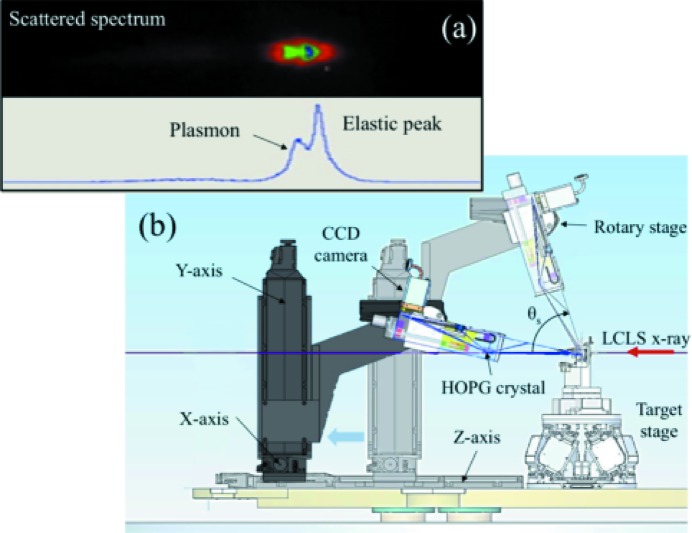
(*a*) Experimental Thomson scattering spectrum of the LCLS seeded beam demonstrating plasmon resolution capabitlities. (*b*) A schematic of the MEC X-ray Thomson scattering spectrometer. The spectrometer is motorized to collect scattering angles θ_s_ between 0 and 90°.

To this aim, MEC developed crystal X-ray scattering spectrometers in a *von Hámos* geometry for the accurate measurement of angle-resolved scattered spectra (see Fig. 5 ▶). The spectrometer detector position can be manually adjusted to accommodate X-ray energies between 4 keV and 8 keV. Motorization of four degrees of freedom allows us to change the scattering angle *in situ* from 0 to 90° with 1° resolution. Cylindrically curved crystals of either highly oriented pyrolytic graphite (HOPG) (Zastrau *et al.*, 2012 ▶) or highly annealed pyrolytic graphite (HAPG) (Zastrau *et al.*, 2013 ▶) are used to efficiently record scattered spectra. The larger mosaic spread of HOPG results in roughly twice the collection efficiency compared with HAPG, while the latter improves the spectral resolution from 30 eV to 9 eV. Fig. 5(*a*) ▶ shows a raw image of the scattered spectrum on an aluminium specimen recorded by the HAPG spectrometer in a forward-scattering geometry. A resolution of 9 eV with seeded LCLS operation at 8 keV clearly distinguishes the plasmon feature down-shifted from the elastic peak by approximately 20 eV. With dynamic shock compression, the shift of the plasmon peak results in a direct measurement of electron density (Fletcher *et al.*, 2013 ▶).

### 3.3. X-ray diffraction of shocked materials   

**Figure 6:**
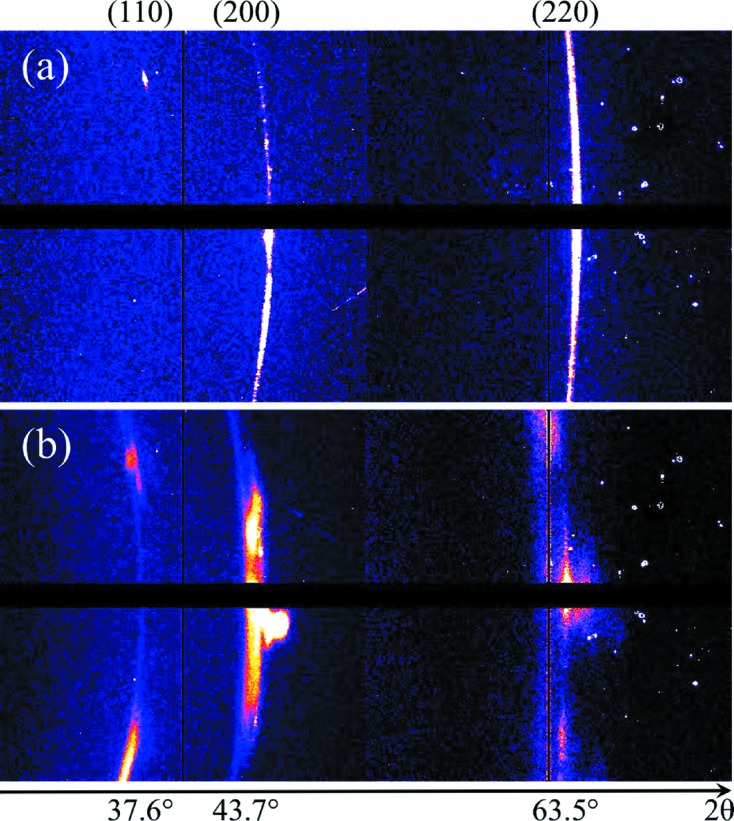
X-ray diffraction of an aluminium sample. Debye–Scherrer rings of (*a*) unshocked sample and (*b*) sample 20 ns after shock loading. Lattice decompression and growth of crystallites are observed.

The opportunity to use an X-ray free-electron laser for investigating phase transitions of materials subjected to high-pressure shocks was recognized early on (Nagler *et al.*, 2007 ▶). Such high-pressure shocks are routinely generated using high-energy laser systems in facilities around the world. Using diffraction from laser-produced X-rays to probe the changes in the microscopic structure of the material has been used very successfully over the last two decades on single crystals (see Loveridge-Smith *et al.*, 2001 ▶; Kalantar *et al.*, 2005 ▶; Jensen & Gupta, 2008 ▶; Murphy *et al.*, 2010 ▶; Suggit *et al.*, 2012 ▶, and references therein). However, the investigation of polycrystalline materials with laser-produced X-ray sources is limited in applicability due to the divergent nature of these sources and the quality of the data that can be achieved. In contrast, the almost perfect collimation of LCLS in combination with its high photon flux make it an excellent source for such research (Milathianaki *et al.*, 2013 ▶). The combination of LCLS with the laser system that is capable of driving Mbar shocks through solid and co-located diagnostics (such as VISAR, XRTS) provides a unique platform for this research. Diffraction of the sample under test is recorded with CSPAD detectors (MEC has both CSPAD-140k and CSPAD-560k) that have been developed by Cornell University and SLAC (Blaj *et al.*, 2015 ▶). In Fig. 6 ▶ we show the diffraction recorded on a CSPAD-140k, from a structured Al foil of thickness 100 µm. Fig. 6(*a*) ▶ shows the unshocked foil. Fig. 6(*b*) ▶ shows the diffraction 20 ns after optical beam has impinged on the target, showing the growth of grains along favorable orientations as well as clear evidence of lattice decompression under shock release. The method can be used to investigate phase transitions, compression and strength of materials with unprecedented temporal resolution.

## 4. Conclusion   

The LCLS beam provides unprecedented brilliance in the hard X-ray part of the spectrum, ideal for experiments in extreme conditions. The MEC instrument complements the LCLS beam with high-power laser and dedicated diagnostics, and as such provides a new exciting platform to perform science in extreme conditions. More details about the MEC instrument can be found on the following website: http://lcls.slac.stanford.edu/mec.

## 5. Facility access   

LCLS instruments are open to academia, industry, government agencies and research institutes worldwide for scientific investigations. There are two calls for proposals per year and an external peer-review committee evaluates proposals based on scientific merit and instrument suitability. Access is without charge for users who intend to publish their results. Prospective users are encouraged to contact instrument staff members to learn more about the science and capabilities of the facility, and opportunities for collaboration.

## Figures and Tables

**Table 1:** X-ray parameters and capabilities of the MEC instrument

Instrument name	MEC
Mirrors, incidence angle	3 SiC on Si, 1.32mrad
Monochromaticity ( 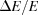 )†	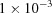 (SASE), 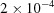 (seeded)
Energy range (keV)	2.5 to 11.0 (fundamental)
Unfocused beam size (m)	1000 at 8keV
Focused beam size (m)	2.0 to 100.0
Focusing optics	Be lenses, 1D and 2D focusing
Flux (photonspulse^1^)	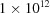 (fundamental‡)
Pulse length (fs)	5200
Repetition rate (Hz)	120, 60, 30, 10, 5, 1, on demand
Optical laser parameters	Ti:sapphire laser: 1J, 50fs, 5Hz, 800nm
	Glass laser: 2 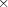 25J, 2100ns, 527nm, 1shot every 7min
Standard detectors	CSPAD-140k, CSPAD-560k,
	Princeton MTE-2048 and MTE-1300
Standard diagnostics	VISAR, FDI, X-ray Thomson scattering spectrometers
	XUV spectrometer, phase-contrast imaging diagnostic

† Typical single-shot value.

‡ Excluding beamline and instrument transmission.
